# HIV-related stigma, depression and suicidal ideation among HIV-positive MSM in China: a moderated mediation model

**DOI:** 10.1186/s12889-023-17047-y

**Published:** 2023-10-27

**Authors:** Jiaqi Fu, Xu Chen, Zhenwei Dai, Yiman Huang, Weijun Xiao, Hao Wang, Mingyu Si, Yijin Wu, Ling Zhang, Shu Jing, Xin Liu, Fei Yu, Guodong Mi, Xiao-You Su

**Affiliations:** 1 School of Population Medicine and Public Health, Chinese Academy of Medical Sciences & Peking Union Medical College, Beijing, China; 2 Danlan Public Welfare, Beijing, China

**Keywords:** MSM, HIV/AIDS, Suicidal ideation, HIV-related stigma, Depression

## Abstract

**Background:**

As the HIV epidemic among MSM in China continues, Chinese men who have sex with men (MSM) face various mental health difficulties, including suicide ideation, depression, and stigma. The current study aims to assess the mechanisms between HIV-related stigma, depression, and suicidal ideation among MSM in China.

**Methods:**

This national cross-sectional study was completed on the geosocial networking application (GSN) app, Blued, from December 2020 to March 2021. We used the HIV Stigma Scale and the Center for Epidemiologic Studies Depression Scale (CES-D_10_) to measure HIV stigma and depression, respectively. Suicidal ideation was measured by the suicidal ideation-related item. Descriptive analyses, logistic regression, and structural equation modeling (SEM) were used for data analysis.

**Results:**

A total of 244 HIV-positive MSM were included in the analysis. The mediation model revealed that the direct pathway of perceived HIV-related stigma on suicidal ideation was significant (standardized pathway coefficient = 0.07), and the indirect pathway of perceived HIV-related stigma on suicidal ideation via depression was also significant (standardized pathway coefficient = 0.04). There was a partial mediating effect of depression in the association between perceived HIV-related stigma and suicidal ideation.

**Conclusions:**

Our study found that both perceived HIV-related stigma and depression were associated with suicidal ideation among HIV-positive MSM in China, and that depression could serve as a mediator between HIV-related stigma and suicidal ideation. Targeted interventions regarding HIV-related stigma and depression should be taken into account to reduce suicidal ideation among HIV-positive MSM in China.

## Background

HIV/AIDS has caused a noticeable crisis in global public health and a significant disease burden, as well as serious economic, educational, and social consequences. By 2022, there were about 38.4 million people living with HIV in the world, and 1.5 million new HIV infections were diagnosed in 2021 [1]. Except in sub-Saharan Africa, men who have sex with men (MSM) account for 45% of new HIV infections [1]. A similar situation exists in China, where homosexual transmission accounted for 23.3% of new HIV infections reported in 2020 [2]. In addition, according to data from the Chinese Center for Disease Control and Prevention, the prevalence of HIV infection among MSM in China is on the rise, from 6.9% to 2019 to 8.0% in 2020 [3, 4].

Coupled with the growing HIV epidemic among MSM in China, several studies indicate that Chinese MSM confront many mental health problems, including suicide ideation, depression, and stigma [5–7]. Suicide, one of the most prominent causes of death among MSM, has emerged as a public health concern [8]. In Canada, the number of deaths caused by suicide is higher than the number of deaths caused by HIV infection among HIV-positive MSM [9]. In China, a significant surge in suicidal behavior among MSM has also been widely noticed [10]. The paths to suicide are complicated. Suicide behavior progresses through three stages: suicidal ideation, suicide attempt, and suicide completion [11]. Suicidal ideation, defined as thinking about, considering or planning suicide, is considered a precursor to suicidal behavior and an important predictor of suicide attempts [12–14]. A meta-analysis suggested that the risk of suicide is 4.17 times higher in people with suicidal ideation than in those without suicidal ideation [13]. And HIV-positive MSM were at higher risk of suicidal ideation than HIV-negative MSM [15]. In an HIV Outpatient Study (HOPS) conducted in the USA from 2000 to 2017, the MSM accounted for 54% of all HIV-positive participants who had suicidal ideation [16]. A systemic review in 2020 reported that the lifetime prevalence of suicidal ideation among MSM in China was 21.2%, much higher than the prevalence in the general public (3.9%) and male population (6.4%) [6, 17, 18]. Given the high prevalence of suicidal ideation and the essential importance of suicidal ideation in the suicidal phase, assessing suicidal ideation among HIV-positive MSM is critical.

The prevalence of suicidal ideation among MSM may differ by age, educational level, economic level, high-risk sexual behaviors, and attitudes towards sexual minorities [8, 11]. In addition, the previous studies also demonstrated that suicidal ideation was associated with depression and HIV-related stigma among HIV-positive MSM [19]. Compared to MSM without HIV, MSM living with HIV were more likely to be depressed [20]. A meta-analysis reported that the prevalence of depression among MSM living with HIV was 43% [21]. Depression is not only associated with suicidal ideation but may also cause the transmission of HIV. For example, MSM who suffer from depressive symptoms are more likely to engage in inconsistent condom use during anal sex [22]. Therefore, regular screening for depression and effective treatments should be developed urgently for HIV-positive MSM [21].

Before 2001, homosexuality was classified as a psychological disorder in China according to the Chinese Classification and Diagnostic Criteria of Mental Disorders [23]. Although this diagnosis had been removed from the criteria, the general public and mental healthcare providers continued to believe that same-sex attraction is psychopathic [22]. And in Asian cultures, heterosexual marriage and the continuation of life are conventionalized responsibilities [24, 25]. Although surrogacy makes it possible for MSM to be parents, it is illegal in China [26]. Therefore, stigma associated with fertility stress is still pervasive in Chinese MSM society [27]. Notably, HIV-positive MSM face extra stigma and marginalization caused by HIV infection compared with HIV-negative MSM [28]. HIV-related stigma and discrimination can occur at a variety of levels, including among individuals, family members, peers, organizations, communities, and public policy [29]. There are many problems that can arise from the stigma of HIV-positive MSM. For example, MSM populations are at high risk for suicidal ideation, which may be associated with discrimination and stigma [15, 30, 31]. A study conducted in Chengdu among HIV-positive MSM found that HIV stigma is associated with depression [32].

To improve the quality of life of HIV-positive MSM, it is essential to identify the risk and protective factors as well as their potential influencing paths on suicidal ideation among HIV-positive MSM, and to provide cost-effective means for addressing both their psychological functioning and HIV risk. The present study is aimed at investigating the prevalence of suicidal ideation among HIV-positive MSM in China, and exploring the association between depression, perceived HIV stigma, and suicidal ideation through a structural equation modeling approach. The mediation effect of depression between HIV-related stigma and suicidal ideation will be tested. This study is expected to provide references for the prevention and intervention of suicidal ideation among HIV-positive MSM.

## Method

### Participants

This cross-sectional study was conducted from December 2020 to March 2021. The patients were eligible if they were: (1) aged 18 or older; (2) born biologically male; (3) HIV testing result was positive; (4) had a registered account with the Blued app; and (5) completed the online informed consent. The exclusion criteria were: (1) being below 18 years old; (2) never having taken an HIV test or having a negative result; and (3) being unable to complete online informed consent. Finally, a total of 244 HIV-positive MSM were included in the analysis.

### Survey procedure

This study was completed on China’s largest geosocial networking application (GSN) app, Blued, which is now a popular social media platform among MSM [33, 34]. The survey invitations were sent to every Blued user in mainland China. Interested participants will click on the invitation to enter the interface for informed consent. In the informed consent process, participants will be informed about the content of this study. Individuals who agreed to participate in the survey study completed the questionnaire anonymously. The study was approved by the Ethics Committee of Danlan Beijing Media Limited on May 20, 2020 (Number: DLIRB202005-01).

### Measures

#### Sociodemographic characteristics

The sociodemographic characteristics of HIV-positive MSM include age, ethnicity, education level, marital status, occupation, the average monthly income in the past year, self-reported sexual orientation, whether they had sex with a male in the last 6 months, whether they had sex with a female in the last 6 months, and rush popper use in the last 6 months.

#### Psychosocial variables

Perceived HIV stigma was measured by the 9-item HIV stigma scale [35]. With each item, participants were required to respond and rate their stigma experience using a 4-point Likert scale (a lot = 1, some = 2, a little = 3, none = 4). The total score was computed for analysis, with higher scores indicating a more severe HIV stigma. The Cronbach’s alpha coefficient was 0.814 for this study.

In our study, depressive symptoms were measured with the Center for Epidemiologic Studies Depression Scale (CES-D_10_). The CES-D_10_ is a 10-item scale developed by Radloff in 1977 to measure depressive symptoms within the past week [36]. The response format ranges from 0 to 3, with 0 representing rarely or never (less than 1 day), 1 representing some or a little of the time (1–2 days), 2 representing occasionally or a moderate amount of the time (3–4 days), and 3 representing most or all of the time (5–7 days). The total score ranged from 0 to 30, with a score of 10 or higher indicating the presence of depressive symptoms. More severe depression was indicated by higher total scores. The Cronbach’s alpha was 0.927 in this study.

About suicidal ideation, the participants were asked, “During the last 6 months, have you ever had thoughts of taking your own life, even if you would not actually do it?“ (The response options were 1 = yes and 2 = no.)

### Statistical analysis

A univariate logistic regression analysis was performed to examine statistically significant associations between variables and suicidal ideation. The variables that were marginally significant with P < 0.10 in the univariate analysis were eligible for entry into the multivariable logistic regression. The χ2 test was applied to explore the relationship between HIV-related stigma and suicidal ideation among HIV-positive MSM. Mediation analysis was performed to assess the mediating role of depression in the association between perceived HIV stigma and suicidal ideation. P value <0.05 was considered statistically significant in this study.

## Results

### Demographic characteristics

A total of 244 HIV-positive MSM met the study inclusion criteria and completed the study. The average age was 34.8 ± 11.227 with 62.7% of the participants being younger than 36 years old. Around 61.6% of the participants had at least a college education. Around 25.8% of the participants reported monthly incomes that were lower than 3000 CNY, 78.7% had a full/part-time job, 80.7% identified themselves as gay. Approximately half (48.4%) of participants had suicidal ideation, and three-fifths (68%) had depression separately. (Table 1)

**Table 1:** Sociodemographic characteristics among HIV-positive MSM (n = 244)

Variables	Frequency	Percentage
**Age(years)**		
18–25	47	19.3%
26–35	106	43.4%
> 36	91	37.3%
**Ethnicity**		
Han	216	88.5%
Others	28	11.5%
**Marital status**		
Unmarried	192	78.7%
Married	52	21.3%
**Work or study status**		
Full/part-time job	192	78.7%
Student	20	8.2%
Unemployed/Retired	32	13.1%
**Education**		
High school or below	95	38.9%
University or above	149	61.1%
**Monthly income(CNY)**		
< 3000	63	25.8%
3000–9999	145	59.4%
> 10,000	36	14.8%
**Sexual orientation**		
Gay	197	80.7%
Other	47	19.3%
**Suicidal ideation**		
No	126	51.6%
Yes	118	48.4%
**Depression**		
No	78	32.0%
Yes	166	68.0%
**HIV stigma**	18.73 ± 7.872	

### Univariate and multivariate factors associated with suicidal ideation

In the univariate logistic analysis, work or study status, monthly income, sex with males in the past 6 months, depression, and HIV stigma were factors associated with suicidal ideation at a p-value of 0.05. For those participants who had depressive symptoms, the odds of suicidal ideation were 4.52 times higher than those without depression (AOR = 4.520, 95%CI: 2.330–8.765 ). Similarly, HIV-positive MSM with a higher level of HIV stigma are more likely to have suicidal ideation (AOR:1.090, 95%CI: 1.046–1.135). (Table 2)

**Table 2:** Univariate and multivariate factors associated with suicidal ideation among HIV-positive MSM (n = 244)

	suicidal ideation														
	All participants					Participants without depression					Participants with depression				
	Yes(n/%)	COR	P	AOR	P	Yes(n/%)	COR	P	AOR	P	Yes(n/%)	COR	P	AOR	P
**Age(years)**															
18–25	27(57.4%)	1				4(40.0%)	1				23(62.2%)	1			
26–35	50(47.2%)	0.661(0.331–1.322)	0.242			7(20.6%)	0.389(0.086–1.767)	0.221			43(59.7%)	0.903(0.400-2.038)	0.805		
> 36	41(45.1%)	0.607(0.298–1.236)	0.169			6(17.6%)	0.321(0.069–1.502)	0.149			35(61.4%)	0.968(0.413–2.270)	0.941		
**Ethnicity**															
Han	106(49.1%)	1				17(23.9%)					89(61.4%)	1			
Others	12(42.9%)	0.778(0.352–1.723)	0.536			0					12(57.1%)	0.839(0.332–2.119)	0.71		
**Marital status**															
Unmarried	93(48.4%)	1				14(24.6%)	1				79(58.5%)	1			
Married	25(48.1%)	0.986(0.534–1.820)	0.963			3(14.3%)	0.512(0.131-2.000)	0.336			22(71.0%)	1.733(0.742–4.045)	0.204		
**Work or study status**															
Full/part-time job	83(43.2%)	1		1		12(17.4%)	1		1		71(57.7%)	1			
Student	13(65.0%)	2.439(0.932–6.384)	0.069	2.314(0.675–7.938)	0.182	2(66.7%)	9.500(0.796-113.426)	0.075	2.989(0.164–54.415)	0.459	11(64.7%)	1.343(0.467–3.864)	0.585		
Unemployed/Retired	22(68.8%)	2.889(1.298–6.431)	0.009	1.613(0.608–4.278)	0.337	3(50.0%)	4.750(0.853–26.450)	0.075	1.156(0.119–11.204)	0.900	19(73.1%)	1.988(0.778–5.076)	0.151		
**Education**															
High school or below	48(50.5%)	1				10(33.3%)	1		1		38(58.5%)	1			
University or above	70(47.0%)	0.868(0.518–1.452)	0.589			7(14.6%)	0.341(0.113–1.030)	0.056	0.443(0.121–1.623)	0.219	63(62.4%)	1.178(0.623–2.227)	0.614		
**Monthly income(CNY)**															
< 3000	38(60.3%)	1		1		8(57.1%)	1		1		30(61.2%)	1			
3000–9999	66(45.5%)	0.550(0.301–1.003)	0.051	1.057(0.465–2.403)	0.895	9(17.0%)	0.153(0.043–0.551)	0.004	0.212(0.040–1.121)	0.068	57(62.0%)	1.031(0.506–2.103)	0.932		
> 10,000	14(38.9%)	0.419(0.181–0.969)	0.042	0.675(0.235–1.940)	0.465	0					14(56.0%)	0.806(0.304–2.141)	0.665		
**Sexual orientation**															
Gay	93(47.2%)	1				13(21.7%)	1				80(58.4%)	1			
Other	25(53.2%)	1.271(0.672–2.404)	0.461			4(22.2%)	1.033(0.290–3.677)	0.96			21(72.4%)	1.870(0.774–4.520)	0.164		
**Had sex with male in the last 6 months**															
No	43(58.9%)	1		1		5(29.4%)	1				38(67.9%)	1			
Yes	75(43.9%)	0.545(0.313–0.950)	0.032	0.805(0.424–1.527)	0.506	12(19.7%)	0.588(0.174–1.990)	0.393			63(57.3%)	0.635(0.323–1.249)	0.188		
**Had sex with female in the last 6 months**															
No	109(47.6%)	1				14(19.2%)	1		1		95(60.9%)	1			
Yes	9(60.0%)	1.651(0.569–4.791)	0.356			3(60.0%)	6.321(0.963–41.497)	0.055	5.191(0.638–42.232)	0.124	6(60.0%)	0.963(0.261–3.553)	0.955		
**Consistent condom use in the last 6 months**															
No	60(50.0%)	1				5(17.9%)	1				55(59.8%)	1			
Yes	58(46.8%)	0.865(0.472–1.584)	0.638			12(24.0%)	1.453(0.453–4.656)	0.53			46(62.2%)	1.105(0.590–2.071)	0.755		
**ART**															
No	11(50.0%)	1				1(14.3%)	1				10(66.7%)	1			
Yes	107(48.2%)	0.930(0.387–2.235)	0.872			16(22.5%)	1.745(0.196–15.580)	0.618			91(60.3%)	0.758(0.247–2.329)	0.629		
**Depression**															
No	17(21.8%)	1		1											
Yes	101(60.8%)	5.576(2.995–10.379)	< 0.001	4.520(2.330–8.765)	< 0.001										
**HIV stigma**	21.56 ± 8.227	1.103(1.063–1.145)	< 0.001	1.090(1.046–1.135)	< 0.001	19.06 ± 7.902	1.064(0.992–1.142)	0.083	1.045(0.955–1.143)	0.335	21.98 ± 8.244	1.105(1.053–1.159)	< 0.001		

### The relationship between HIV stigma and suicidal ideation among HIV-positive MSM

Compared with MSM without suicidal ideation, participants who had suicidal ideation felt more severe estrangement from people (19.5% vs. 7.9%), blame from people (20.3% vs. 6.3%), estrangement from family members (15.3% vs. 4.8%) because of their HIV status. Among HIV-positive MSM who had suicidal ideation, they felt it was more difficult to get along with people (30.5% vs. 7.1%) as well as show their advantages (22.9% vs. 9.5%), and they had to be kept away from children (26.3% vs. 7.1%). Besides, HIV-positive MSM with suicidal ideation were more worried about their HIV status affecting their family members’ employment (21.2% vs. 5.6%) and education rights (35.6% vs. 12.7%). A similar situation also existed among participants with and without depression, respectively. (Table 3)

**Table 3:** The relationship between HIV stigma and suicidal ideation among HIV-positive MSM. (n = 244)

	All participants				Participants with depression				Participants without depression			
	Yes(%)	No(%)	χ^2^/t	p	Yes(%)	No(%)	χ^2^/t	p	Yes(%)	No(%)	χ^2^/t	p
**1.Because of my HIV status, I feel estranged by people around me.**												
A lot	23(19.5%)	10(7.9%)	24.382	< 0.001	19(18.8%)	6(9.2%)	10.543	0.014	4(23.5%)	4(6.6%)	5.518	0.138
Some	22(18.6%	15(11.9%)			20(19.8%)	8(12.3%)			2(11.8%)	7(11.5%)		
A little	47(39.8%)	36(28.6%)			42(41.6%)	24(36.9%)			5(29.4%)	12(19.7%)		
None	26(22.0%)	65(51.6%)			20(19.8%)	27(41.5%)			6(35.3%)	38(62.3%)		
**2.Because of my HIV status, I feel blamed by people around me.**												
A lot	24(20.3%)	8(6.3%)	25.395	< 0.001	20(19.8%)	3(4.6%)	14.812	0.002	4(23.5%)	5(8.2%)	4.922	0.178
Some	23(19.5%)	20(15.9%)			20(19.8%)	14(21.5%)			3(17.6%)	6(9.8%)		
A little	41(34.7%)	29(23.0%)			37(36.6%)	17(26.2%)			4(23.5%)	12(19.7%)		
None	30(25.4%)	69(54.8%)			24(23.8%)	31(47.7%)			6(35.3%)	38(62.3%)		
**3.Because of my HIV status, I feel estranged by my family members.**												
A lot	18(15.3%)	6(4.8%)	13.514	0	16(15.8%)	1(1.5%)	10.994	0.012	2(11.8%)	5(8.2%)	7.317	0.062
Some	17(14.4%)	11(8.7%)			15(14.9%)	6(9.2%)			2(11.8%)	5(8.2%)		
A little	31(26.3%)	28(22.2%)			25(24.8%)	22(33.8%)			6(35.3%)	6(9.8%)		
None	52(44.1%)	81(64.3%)			45(44.6%)	36(55.4%)			7(41.2%)	45(73.8%)		
**4.Because of my HIV status, I feel it is very hard for my family members to get married.**												
A lot	55(46.6%)	40(31.7%)	7.243	0.07	49(48.5%)	20(30.8%)	5.535	0.137	6(35.3%)	20(32.8%)	2.554	0.466
Some	14(11.9%)	20(15.9%)			10(9.9%)	10(15.4%)			4(23.5%)	10(16.4%)		
A little	22(18.6%)	22(17.5%)			18(17.8%)	13(20.0%)			4(23.5%)	9(14.8%)		
None	27(22.9%)	44(34.9%)			24(23.8%)	22(33.8%)			3(17.6%)	22(36.1%)		
**5.Because of my HIV status, I feel it is not easy to get along with people around me.**												
A lot	36(30.5%)	9(7.1%)	32.268	< 0.001	35(34.7%)	2(3.1%)	24.634	< 0.001	1(5.9%)	7(11.5%)	4.007	0.261
Some	21(17.8%)	16(12.7%)			18(17.8%)	13(20.0%)			3(17.6%)	3(4.9%)		
A little	44(37.3%)	53(42.1%)			37(36.6%)	34(52.3%)			7(41.2%)	19(31.1%)		
None	17(14.4%)	48(38.1%)			11(10.9%)	16(24.6%)			6(35.3%)	32(52.5%)		
**6.Because of my HIV status, I feel people will no longer see my strong points.**												
A lot	27(22.9%)	11(9.5%)	28.865	< 0.001	26(25.7%)	3(4.6%)	19.237	< 0.001	1(5.9%)	8(13.1%)	8.4	0.038
Some	23(19.5%)	10(7.9%)			20(19.8%)	8(12.3%)			3(17.6%)	2(3.3%)		
A little	34(28.8%)	28(22.2%)			28(27.7%)	19(29.2%)			6(35.3%)	9(14.8%)		
None	34(28.8%)	77(61.1%)			27(26.7%)	35(53.8%)			7(41.2%)	42(68.9%)		
**7.Because of my HIV status, I feel that children are kept away from me by their parents.**												
A lot	31(26.3%)	9(7.1%)	26.496	< 0.001	29(28.7%)	2(3.1%)	24.328	< 0.001	2(11.8%)	7(11.5%)	0.937	0.816
Some	15(12.7%)	18(14.3%)			13(12.9%)	12(18.5%)			2(11.8%)	6(9.8%)		
A little	37(31,6%)	27(21.4%)			32(31.7%)	15(23.1%)			5(29.4%)	12(19.7%)		
None	35(30.0%)	72(57.1%)			27(26.7%)	36(55.4%)			8(47.1%)	36(59.0%)		
**8.Because of my HIV status, I feel it is very hard for my family members to find a job.**												
A lot	25(21.2%)	7(5.6%)	18.801	< 0.001	23(22.8%)	3(4.6%)	11.455	0.01	2(11.8%)	4(6.6%)	2.379	0.498
Some	13(11.0%)	11(8.7%)			11(10.9%)	8(12.3%)			2(11.8%)	3(4.9%)		
A little	31(26.3%)	26(20.6%)			27(26.7%)	16(24.6%)			4(23.5%)	10(16.4%)		
None	49(41.5%)	82(65.1%)			40(39.6%)	38(58.5%)			9(52.9%)	44(72.1%)		
**9.Because of my HIV status, I feel my family members cannot have the same rights to education.**												
A lot	42(35.6%)	16(12.7%)	24.194	< 0.001	39(38.6%)	8(12.3%)	15.555	0.001	3(17.6%)	8(13.1%)	5.16	0.16
Some	13(11.0%)	13(10.3%)			9(8.9%)	7(10.8%)			4(23.5%)	6(9.8%)		
A little	30(25.4%)	28(22.2%)			25(24.8%)	17(26.2%)			5(29.4%)	11(18.0%)		
None	33(28.0%)	69(54.8%)			28(27.7%)	33(50.8%)			5(29.4%)	36(59.0%)		
**Sum**	21.56 ± 8.227	16.09 ± 6.521	5.776	< 0.001	21.98 ± 8.244	16.58 ± 6.169	4.523	< 0.001	19.06 ± 7.902	15.56 ± 6.888	1.795	0.077

### Mediation analysis

**Fig. 1:**
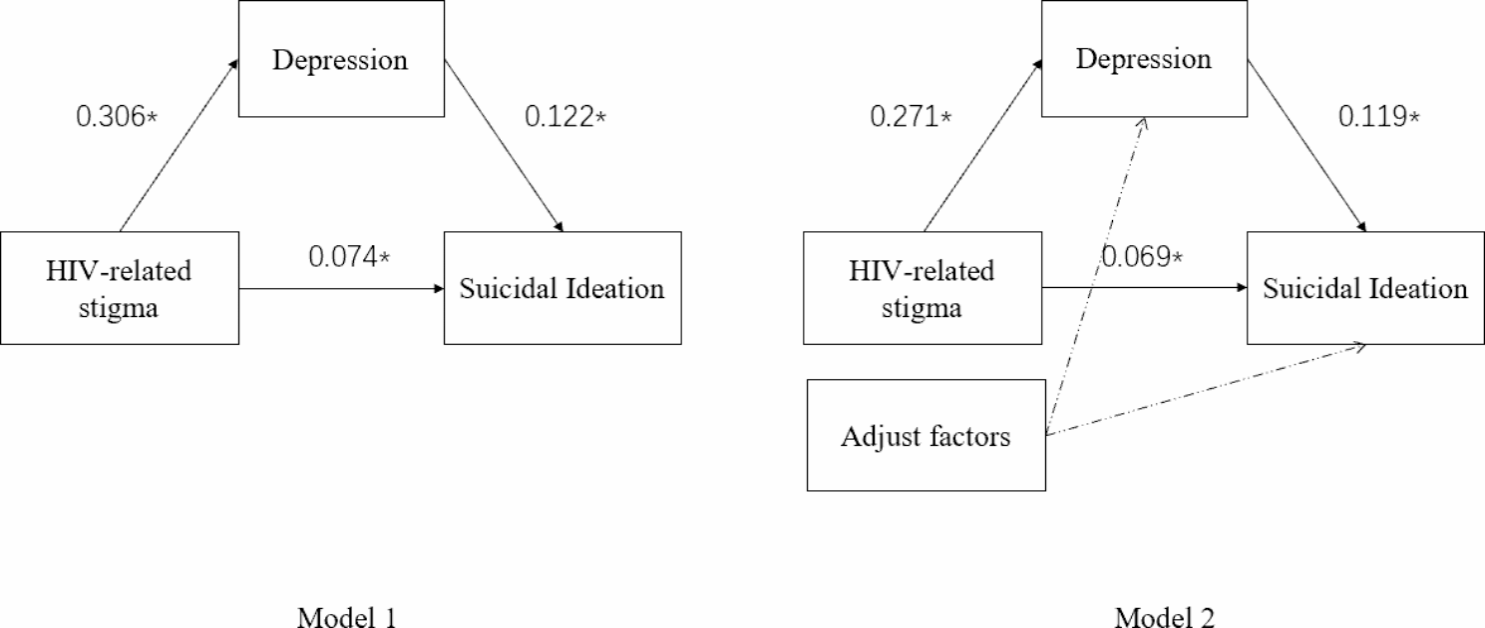
Mediation model. Note. *: P < 0.05. Adjust factors: work or study status, monthly income, had sex with male in the last 6 months

In the mediation model built in this study, depression was positively associated with suicidal ideation (β = 0.122, p < 0.05). HIV-related stigma was positively associated with depression (β = 0.306, p < 0.05) and suicidal ideation (β = 0.074, p < 0.05). After adjusting work or study status, monthly income, and having sex with at least one male in the last 6 months, the mediating effect remained. Details were shown in Fig. 1; Table 4.

**Table 4:** Results of mediating effects

Item		Model 1	Model 2
c	Total effect	0.111*	0.101*
a		0.306*	0.271*
b		0.122*	0.117*
a*b	Mediating effect value	0.037*	0.032*
a*b	(Boot SE)	0.011	0.011
a*b	(95% BootCI)	(0.019–0.063)	(0.015–0.057)
c’	Direct mediating effect	0.074*	0.069*
Test result		Partially mediated	Partially mediated
Effect account		33.3%	31.7%

Note: S.E.: Standard error *p < 0.05

Model 1: No adjustment

Model 2: Adjust for work or study status, monthly income, had sex with male in the last 6 months

## Discussion

In this cross-sectional study, a mediation model was built to test the association between perceived HIV-related stigma, depression, and suicidal ideation among HIV-positive MSM in China. In our results, HIV stigma and depression were both associated with suicidal ideation among Chinese HIV-positive MSM, and depression may have mediated the association between HIV stigma and suicidal ideation.

In our study, the rate of suicidal ideation among HIV-positive MSM was 48.4%, which was higher than the previous study conducted in Anhui in 2015 (31.0%) and Changsha in 2022 (32.8%), but similar to the research conducted in Chengdu in 2018 (48.0%) [19, 37, 38]. A possible reason is that the participants in our study were recruited during the COVID-19 pandemic. Compared to the general population, HIV-infected MSM were more vulnerable to mental health problems during the COVID-19 pandemic [39]. A study from the Republic of Ireland found that 75% of MSM reported that their mental health worsened during the COVID-19 pandemic because of the closure of MSM venues and limits on socializing, leading to a decrease in well-being and even committing suicide [40]. We suspected that the MSM population in China may be similarly affected for the same reason. On the other hand, HIV-positive MSM face dual pressures, including sexual minority identification and discrimination against individuals living with HIV, both in China and in other countries [22, 41]. These pressures may gradually worsen their survival situation and lead to the occurrence of suicidal ideation [37]. Therefore, it is necessary to take measures to strengthen identity recognition and reduce HIV-stigma, thereby reducing the prevalence of suicidal ideation among HIV-positive MSM. A qualitative study conducted in Uganda showed that increasing knowledge of specific care concerning MSM among healthcare providers in general healthcare settings to mitigate negative experiences of MSM is helpful to identity recognition [42]. Another cross-sectional survey conducted in Changsha pointed out that increasing awareness of mental health conditions and strengthening self-regulation skills are useful to avoid HIV-related stigma and further reduce suicidal ideation among HIV-positive MSM [37]. In addition, during an epidemic like COVID-19, it is particularly important for the government and relevant agencies to provide online and offline HIV care support interventions to HIV-positive MSM [43, 44].

Around 68.0% of HIV-positive MSM had depressive symptoms in our study, which was higher than the results of previous studies conducted in the similar population in the United Kingdom in 2018 (57.9%), Los Angeles in 2017 (60%), and Nanjing in 2016 (38.6%) [28, 45, 46]. As a vulnerable population under the mainstream of heterosexual culture, MSM often feel guilty, have low self-esteem, and fear discrimination, which may eventually lead to depression [47]. Previous researchers also noted that the HIV infection, which is believed to be life-threatening by some of them could leads patients into distressing psychological and emotional states of mind and may further lead to depression [48]. Some HIV-infected individuals even think that their lives are not worth living anymore due to multiple reasons and pressures [49]. For example, some patients believe that HIV is out of control, chronic, and has serious consequences for which there is no treatment, which will increase their suicidal ideation [38]. A meta-analysis showed that the frequency of major depressive disorder was nearly two times higher among HIV-positive MSM than among HIV-negative MSM [50]. Another possible reasons for this discrepancy may be different measurement scale for depression. In other studies, depression was measured by the 14-item Hospital Anxiety and Depression Scale (HADS), while our research used CES-D_10_ to measure depression [45]. The CES-D is usually used as a screen tool in clinical practice and has high sensitivity, which may have led to the higher depression prevalence found in our study [51]. Another reason worth mentioning is that participants in our study might have difficulty gaining access to their antiretroviral medications during the COVID pandemic, and these disruptions to HIV treatment and care services may exacerbate the existing mental health burdens faced by this population [52]. Steven’s previous research shows that HIV-positive MSM with depression were demonstrated to have higher odds of engaging in sexual transmission risk behaviors like condomless sex and further increase the likelihood of transmitting HIV [53]. On the other hand, depression among HIV-positive MSM could have a negative influence on their adherence to antiretroviral therapy [54]. Therefore, it is necessary to monitor their mental status frequently and alleviate their possible depression among HIV-positive MSM. Health care providers treating HIV-positive patients should regularly screen for depression among HIV-positive MSM [46]. Recently, Li et al. reported an innovative approach for the monitoring of depressive emotions among the MSM population via social media [55]. It is hopeful to monitor depression among MSM by means of networking and take measures to deal with psychological problems through social media in the future.

The level of HIV-related stigma in our research was generally consistent with the findings of a previous study in Beijing [56]. And in our study, MSM with a higher level of stigma, including concerns about family members’ working and educational rights as well as their relationship with people around them because of their own HIV status were more likely to have suicidal ideation. This is consistent with previous studies conducted in Anhui that found the likelihood of suicidal ideation is significantly associated with HIV-stigma [19]. In the context of traditional sexual values, homosexuality is unacceptable to the majority of Chinese people [57]. Besides the above pressure, HIV-positive MSM are often considered to be infectious [56]. Thus, in most situations, in order to prevent social rejection, HIV-positive MSM will not disclose their HIV status to avoid being isolated from participating in social events [58]. In turn, MSM who are unwilling to disclose their HIV serostatus could not receive support or acquire others’ care, which further might reduce incentives to cope with psychological adversities [59, 60]. Meanwhile, a high level of HIV-related stigma may have a lot of negative consequences. HIV-positive MSM with high HIV stigma were significantly more likely to engage in unprotected sex while high or intoxicated [61]. Besides, HIV-related stigma could also delay clinic care and increase the chance of HIV transmission for HIV-positive MSM [62]. Hence, various measures should be taken into account to reduce stigma and discrimination. As resilience may function as a buffer against the harmful effect of stigma, it may be one of the feasible solutions that promotes resilience through some interventions like interactive training sessions, group activities, etc. and further decreases stigma among HIV-positive MSM [63, 64]. In addition, because HIV stigma often centers around gossip, social media could spread the information that U = U (undetectable = untransmutable) widely, which could change social attitudes towards the HIV-positive population and tackle stigma and discrimination to some extent [62, 65].

Our findings suggested that depression is associated with suicidal ideation among HIV-positive MSM, which is consistent with the previous studies conducted in Shenzhen, Shenyang, Anhui, et al. [11, 19, 38]. Mu et al. revealed that mental disorders represented by depression constitute the largest reason for suicidal behavior in Chinese MSM [6]. In Yu et al.’s research, MSM with depression experienced more rejection and discrimination, and were more sensitive to their minority status, which caused some Chinese MSM to think about suicide [66]. And in a clinical trial, individuals with depression significantly decreased their suicidal ideation after accepting antidepressant injections [67]. Therefore, it is urgent to take some measures to improve depression among HIV-positive MSM and further reduce suicidal ideation. For example, HIV care providers should routinely assess the mental health of HIV-positive MSM, especially depression [68].

Furthermore, the findings of our study indicated that depression could mediate the association between perceived HIV-related stigma and suicidal ideation among MSM living with HIV in China, that is, HIV-related stigma could not only have a direct effect on suicidal ideation but also have an indirect effect on suicidal ideation through the mediation role of depression, which revealed the pathway of the association between perceived HIV-related stigma and suicidal ideation among HIV-positive MSM in China. The mediation effect could be interpreted to mean that alleviating the HIV-related stigma could help MSM reduce the negative evaluation around them to a certain extent, which makes them less likely to be influenced by discrimination, thus avoiding the development of depression and further reducing their suicidal ideation accordingly [69, 70]. This finding indicated the critical need for structural interventions to reduce HIV-related stigma and depression among MSM living with HIV in China. For example, support group intervention has been shown to be effective in diminishing HIV-related stigma among HIV-positive MSM. Besides, health personnel should be aware of possible depression and even suicidal ideation in HIV-positive MSM who have perceived HIV stigma and pay more attention to the mental health of this population [71]. And a study conducted in Thailand also illustrated that community mobilization activities, which included monthly campaigns, funfairs, and information and educational materials, helped to reduce HIV stigma at the community level [72]. In China, community was seen as an essential part of intervention for MSM. Considering the feasibility and sustainability of the interventions, it is suggested to strengthen the training of peer educators in mental health and integrate mental health interventions into the daily work of peer education, which could provide comfort, education, and a sense of solidarity for MSM [73].

Our study had some limitations. First, it is difficult to avoid social desirability bias and recall bias because the data in our study derive from participants self-report. Second, due to the nature of a cross-sectional investigation, this study cannot establish the causal link between the postulated factors. Third, our participants were recruited from a GSN, therefore, the generalization of our findings to those who do not use Blued is uncertain. We plan to conduct a further survey to explore the relationship between dating app use and mental health among MSM.

## Conclusion

Our study found that both perceived HIV-related stigma and depression were associated with suicidal ideation among HIV-positive MSM in China and that depression could serve as a mediator between HIV-related stigma and suicidal ideation. Targeted interventions regarding HIV-related stigma and depression should be taken into account to reduce suicidal ideation among HIV-positive MSM in China.

## Data Availability

The datasets generated and/or analysed during the current study are not publicly available due to the privacy information of participants but are available from the corresponding author on reasonable request.

## Abbreviations

MSM: Men who have sex with men
GSN: Geosocial networking application
CES-D^10^: Center for Epidemiologic Studies Depression Scale
SEM: Structural equation modeling
HOPS: HIV Outpatient Study
